# Rapid and sensitive determination of residual prion infectivity from prion-decontaminated surfaces

**DOI:** 10.1128/msphere.00504-24

**Published:** 2024-08-27

**Authors:** Sara M. Simmons, Vivianne L. Payne, Jay G. Hrdlicka, Jack Taylor, Peter A. Larsen, Tiffany M. Wolf, Marc D. Schwabenlander, Qi Yuan, Jason C. Bartz

**Affiliations:** 1 Department of Medical Microbiology and Immunology, School of Medicine, Creighton University, Omaha, Nebraska, USA; 2 College of Saint Mary’s, Omaha, Nebraska, USA; 3 Biostatistical Core Facility, Creighton University, Omaha, Nebraska, USA; 4 Department of Veterinary and Biomedical Sciences, College of Veterinary Medicine, University of Minnesota, St. Paul, Minnesota, USA; 5 Minnesota Center for Prion Research and Outreach, College of Veterinary Medicine, University of Minnesota, St. Paul, Minnesota, USA; 6 Department of Veterinary Population Medicine, College of Veterinary Medicine, University of Minnesota, St. Paul, Minnesota, USA; 7 Prion Research Center, Colorado State University, Fort Collins, Colorado, USA; University of Michigan, Ann Arbor, Michigan, USA

**Keywords:** prion disease, prion decontamination, prion surveillance

## Abstract

Prion diseases are untreatable fatal transmissible neurodegenerative diseases that affect a wide range of mammals, including humans, and are caused by PrP^Sc^, the infectious self-templating conformation of the host-encoded protein, PrP^C^. Prion diseases can be transmitted via surfaces (e.g., forceps, EEG electrodes) in laboratory and clinical settings. Here, we use a combination of surface swabbing and real-time quaking-induced conversion (RT-QuIC) to test for residual surface-associated prions following prion disinfection. We found that treatment of several prion-contaminated laboratory and clinically relevant surfaces with either water or 70% EtOH resulted in robust detection of surface-associated prions. In contrast, treatment of surfaces with sodium hypochlorite resulted in a failure to detect surface-associated prions. RT-QuIC analysis of prion-contaminated stainless steel wires paralleled the findings of the surface swab studies. Importantly, animal bioassay and RT-QuIC analysis of the same swab extracts are in agreement. We report on conditions that may interfere with the assay that need to be taken into consideration before using this technique. Overall, this method can be used to survey laboratory and clinical surfaces for prion infectivity following prion decontamination protocols.

**IMPORTANCE:**

Prion diseases can be accidentally transmitted in clinical and occupational settings. While effective means of prion decontamination exist, methods for determining the effectiveness are only beginning to be described. Here, we analyze surface swab extracts using real-time quaking-induced conversion (RT-QuIC) to test for residual prions following prion disinfection of relevant clinical and laboratory surfaces. We found that this method can rapidly determine the efficacy of surface prion decontamination. Importantly, examination of surface extracts with RT-QuIC and animal bioassay produced similar findings, suggesting that this method can accurately assess the reduction in prion titer. We identified surface contaminants that interfere with the assay, which may be found in clinical and laboratory settings. Overall, this method can enhance clinical and laboratory prion safety measures.

## INTRODUCTION

Prion diseases are fatal, neurodegenerative disorders that affect several mammalian species. Human prion diseases include Creutzfeldt-Jakob disease (CJD), Gerstmann-Sträussler-Scheinker disease, fatal familial insomnia, and Kuru (1–4). Prion diseases known to afflict other species include chronic wasting disease in cervids, scrapie in sheep and goats, camel prion disease in dromedary camels, and bovine spongiform encephalopathy (BSE) in cattle (5–9). Prion diseases are caused by the misfolding of the normal cellular form of the prion protein (PrP^C^) into the infectious and pathogenic form (PrP^Sc^) (10–15). PrP^Sc^ converts further PrP^C^ into the pathogenic form eventually leading to neuronal dysfunction and death of the host (16–20). Prion strains are operationally defined by a heritable phenotype of disease that is encoded by strain-specific conformations of PrP^Sc^ (21–23). Mounting evidence indicates that several other protein misfolding neurodegenerative diseases follow the prion paradigm (24–29). While not contagious in a population, these diseases share similarities in prion formation, spread in the nervous system, and strain-specific phenotypes of the disease (25, 27, 30–37).

Prions are highly resistant to common forms of disinfection. Prions and/or PrP^Sc^ are resistant to inactivation by UV light, ionizing radiation, heat, formaldehyde, hydrogen peroxide, and alcohol (38–48). Similarly, multiple system atrophy α-synuclein and amyloid-β prions are resistant to inactivation by formalin fixation (49–51). Recently, it has been shown that Lewy body α-synuclein prions that cause dementia are resistant to inactivation by autoclaving at 121°C (52). Prions can be chemically inactivated with sodium hydroxide, sodium hypochlorite, acidic sodium dodecyl sulfate, hypochlorous acid, and the phenolic compounds Environ LpH and Wexide-128 (53–59). Importantly, prion strain-specific differences in susceptibility to inactivation by both physical and chemical methods have been observed; therefore, the efficacy of antiprion modalities to new prion diseases or strains must be interpreted with caution (56, 60). Strain-specific incomplete inactivation of prions can result in the emergence of a minor prion strain from a mixture (61).

Prions can be transmitted via iatrogenic infection. Iatrogenic infection can arise from prion-contaminated transplant materials or medical equipment. The first reported case of iatrogenic infection originated from a corneal transplant that was derived from a cadaver unknowingly harboring CJD (62). Additionally, iatrogenic CJD can occur after the transplantation of CJD-contaminated human growth hormone or dura mater (63–73). Iatrogenic Alzheimer’s disease in recipients of growth hormone derived from pituitary glands that contain Aβ prions has been observed (36, 74, 75). Cases of iatrogenic CJD transmission have been reported following the use of contaminated neurosurgical tools involved in procedures such as brain biopsies and tumor removals on a patient later diagnosed with CJD (76–80).

Occupational prion transmission has been reported. The first account of an occupational prion transmission occurred in a laboratory worker who was exposed to brain material from humanized transgenic mice infected with sheep-adapted BSE, likely from a puncture wound from forceps (81). A second suspect case of occupational CJD attributed to laboratory transmission was reported in 2021 resulting in a moratorium on prion research in France (82). The reported transmission of non-PrP-based prions from the iatrogenic transmission and laboratory studies, in conjunction with PrP and some non-PrP prions’ unusual resistance to inactivation, highlights the potential for prion transmission under occupational and medical settings (83).

Prion decontamination efficacy has been measured through bioassay of treated samples. This can be accomplished by animal bioassay of treated tissue homogenates or prion-coated stainless steel wires (84, 85). While effective, this process is costly and time-consuming (86, 87). The scrapie cell assay (SCA) is as sensitive as animal bioassay, more precise, and can be completed in a fraction of the time (88). The SCA can accurately measure prions on the surface of stainless steel wires to determine the efficacy of prion decontamination methods (89). A weakness of this method is only a limited number of prion strains are compatible with the SCA, and the method is not able to assess for prions on working surfaces in laboratory or clinical settings. Ultra-sensitive prion detection assays, such as real-time quaking-induced conversion (RT-QuIC), have allowed for the amplification and detection of minute amounts of PrP^Sc^ and the prion forms of synuclein, tau and Aβ (90–95). Recent work indicates that PrP, α-synuclein, and tau prions can be detected from media that are applied to a surface (96). Here, we investigate if the combination of RT-QuIC coupled with the newly developed swabbing methodology provides a practical approach to assess surface decontamination efficacy in laboratory and clinical settings (97).

## RESULTS

### Efficient recovery of prions applied to laboratory surfaces

**Fig 1:**
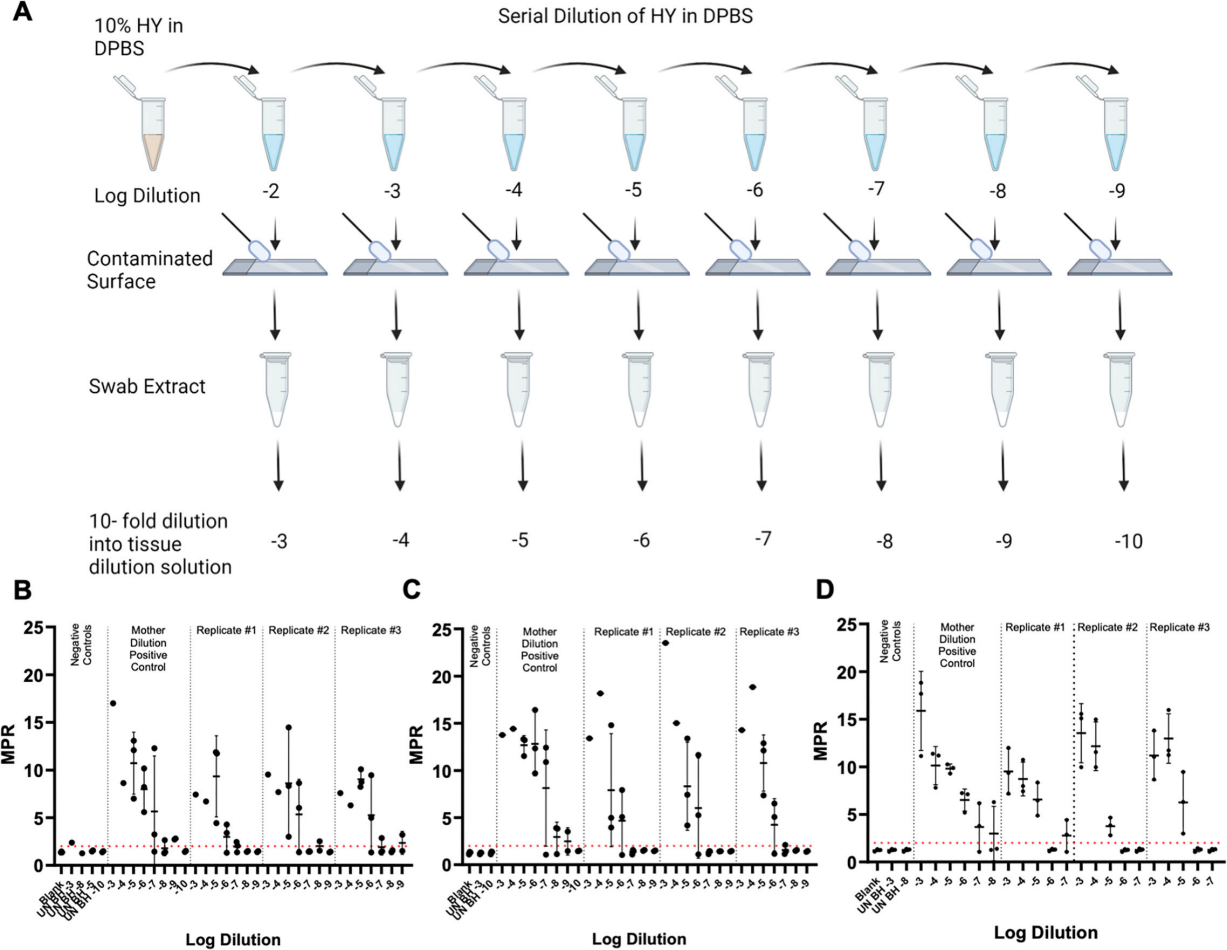
Effective swabbing recovery of prions applied to laboratory surfaces. (**A**)Vertical dilution method surface contamination and swabbing methodology of contaminated laboratory surfaces. Created with BioRender.com. (**B**)RT-QuIC detection for glass slide surface-recovered HY prions. (**C**)RT-QuIC detection for stainless steel surface-recovered HY prions. (**D**)RT-QuIC detection for laboratory benchtop surface-recovered HY prions. Negative plate controls include blank (tissue dilution solution) and uninfected brain homogenate. A positive fluorescence threshold (illustrated by the red line) was determined to be at 2. The maxpoint ratio reported is the ratio of the maximum fluorescence to the initial fluorescence reading obtained by the plate reader. Each point represents the average MPR from one biological replicate (mean ± standard deviation).

Each surface type was contaminated with 10-fold serial dilutions of hyper transmissible mink encephalopathy (HY TME)-infected brain homogenate in Dulbecco’s phosphate-buffered saline (DPBS) (mother dilutions), and contaminated surfaces were allowed to dry for 24 hours prior to swabbing, extraction, and RT-QuIC analysis (Fig. 1, panel A). Negative control swab extracts failed to yield positive readings, in terms of maxpoint ratio (MPR) above the determined positive threshold of 2. In some instances, however, positive signals were observed in one out of four technical well replicates for one technical negative swab extract replicates. When comparing the SD_50_ (seeding dose that resulted in 50% positivity for tested samples determined by RT-QuIC end-point dilutions, Table 1) of swabbing-recovered HY TME to the original HY TME mother dilutions applied to the surface, there was an approximate loss of 1.1 logs for glass surfaces (Fig. 1, panel B), 1.6 logs for stainless steel surfaces (Fig. 1, panel C), and 2 logs for benchtop surfaces (Fig. 1, panel D), respectively.

**TABLE 1:** Comparison of log SD_50_/g of brain homogenate, determined by RT-QuIC end-point dilutions, of prion-contaminated surface swabbing recovered HY

Surface	Biological replicate	Mother dilution	Technical replicate #1	Technical replicate #2	Technical replicate #3	Technical replicate^*a*^
Glass slide	#1	10.53	8.93	9.03	9.20	9.05 ± 0.11
	#2	9.33	8.78	9.17	8.92	8.96 ± 0.16
	#3	9.70	8.17	8.03	8.45	8.22 ± 0.17
					
Stainless steel	#1	10.53	9.03	9.20	9.03	9.09 ± 0.08
	#2	10.53	8.70	9.20	8.70	8.87 ± 0.24
	#3	9.20	7.37	7.70	7.37	7.48 ± 0.16
					
Benchtop	#1	10.20	8.33	7.37	8.20	7.97 ± 0.43
	#2	9.20	7.70	7.70	7.37	7.59 ± 0.16
	#3	9.44	7.70	6.33	8.03	7.35 ± 0.74

^*a*^ Average ± SD.

### Residual bleach does not interfere with RT-QuIC detection of surface-recovered HY TME

**Fig 2:**
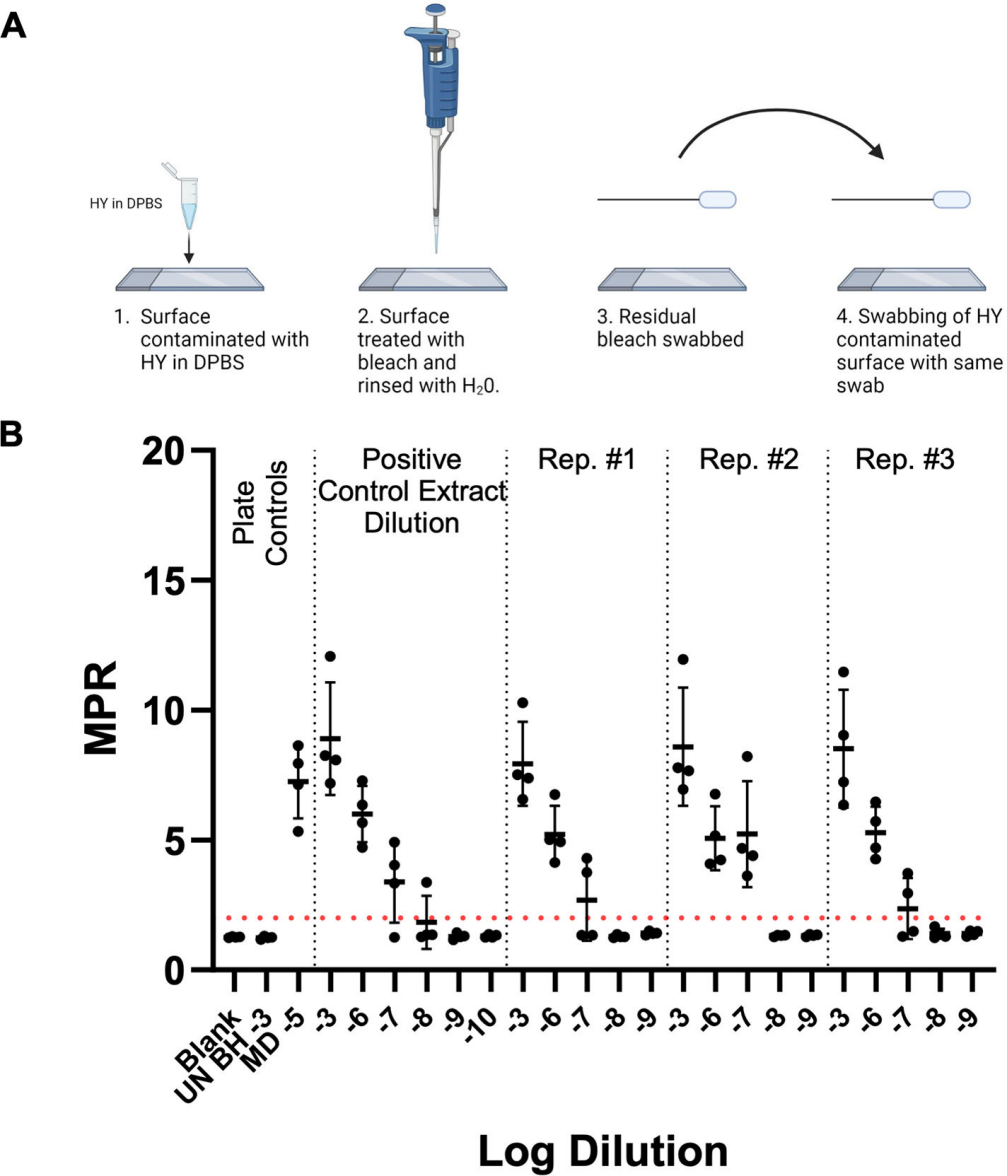
Residual bleach does not interfere with RT-QuIC detection of surface-recovered prions. (**A**)Residual bleach swabbing methodology (Created with BioRender.com.). (**B**)RT-QuIC detection of swab extracts from prion-contaminated surfaces exposed to residual bleach residue. Plate controls included negative plate controls: blank (tissue dilution solution) and uninfected hamster brain homogenate of 10^−3^ and the positive plate control mother dilution 10^−5^. A positive fluorescence threshold (illustrated by the red line) was determined to be at 2. The maxpoint ratio reported is the ratio of the maximum fluorescence to the initial fluorescence reading obtained by the plate reader (mean ± standard deviation). Each experiment was performed at least three times, with similar results obtained from each experiment.

The potential inhibitory role of bleach residue that may be introduced to the RT-QuIC reaction from treated surfaces was investigated. Glass slide surfaces were contaminated with 10^−2^ HY TME-infected brain and were allowed to dry for 24 hours prior to bleach treatment, swabbing, extraction, and RT-QuIC analysis (Fig. 2, panel A). To quantify any potential inhibition imparted by bleach residue, the bleach control swab extracts were run in comparison to a representative swab extract that had not undergone treatment. The SD_50_ was determined and used to assess any loss of detection. Negative plate controls failed to yield MPR above the determined positive threshold of 2. (Fig. 2, panel B). The swabbing of residual bleach, followed by swabbing of an additional surface contaminated with 10^−2^ HY TME-infected brain homogenate in DPBS yielded SD_50_ results, determined by RT-QuIC end-point dilutions, that were within 0.2 log when compared to 10^−2^ HY brain homogenate (BH) swab extract samples alone (Table 2). The results of this experiment illustrate that residual bleach, which may be present on a treated surface following thorough rinsing with water, does not inhibit the RT-QuIC reaction. Overall, the reduction of RT-QuIC seeding activity with bleach treatment is a result of decontamination.

**TABLE 2:** RT-QuIC detection (log SD_50_/g of brain homogenate as determined by end-point dilutions) of HY recovered from relevant surfaces treated with various disinfectants

Surface	Biological replicate	Technical replicate	Treatment				
			None	H_2_O	70% EtOH	Bleach	Bleach control
Glass slide	1	a	10.20	10.20	9.20	MPR< 2	10.70
		b	10.47	9.37	9.37	MPR< 2	10.70
		c	11.20	10.20	9.70	MPR< 2	10.20
	2	a	10.93	9.20	9.53	MPR< 2	9.70
		b	10.37	9.37	9.20	MPR< 2	10.20
		c	10.53	8.20	10.03	MPR< 2	9.70
	3	a	9.20	8.33	8.20	MPR< 2	9.47
		b	9.70	8.93	9.33	MPR< 2	9.53
		c	9.20	8.20	9.20	MPR< 2	10.20
	Avg± SD		10.20 ± 0.66	9.11 ± 0.73	9.31 ± 0.47	MPR< 2	10.04 ± 0.44^*a*^
Stainless steel	1	a	10.53	10.32	9.47	MPR< 2	N/A^*b*^
		b	10.37	9.70	10.20	MPR< 2	N/A
		c	9.93	9.70	9.37	MPR< 2	N/A
	2	a	9.20	9.30	9.03	MPR< 2	N/A
		b	9.20	8.93	8.70	MPR< 2	N/A
		c	9.37	10.20	9.03	MPR< 2	N/A
	3	a	9.03	10.02	8.20	MPR< 2	N/A
		b	10.03	9.20	9.37	MPR< 2	N/A
		c	8.93	9.20	8.70	MPR< 2	N/A
	Avg± SD		9.62 ± 0.57	9.62 ± 0.46	9.12 ± 0.54	MPR< 2	N/A
Benchtop	1	a	10.93	9.20	10.03	MPR< 2	N/A
		b	10.37	8.70	10.03	MPR< 2	N/A
		c	10.93	9.70	10.20	MPR< 2	N/A
	2	a	10.37	10.03	10.37	MPR< 2	N/A
		b	11.37	9.93	10.20	MPR< 2	N/A
		c	10.70	10.03	10.20	MPR< 2	N/A
	3	a	10.37	8.37	9.37	MPR< 2	N/A
		b	10.37	8.93	10.03	MPR< 2	N/A
		c	10.93	10.03	9.93	MPR< 2	N/A
	Avg± SD		10.70 ± 0.34	9.44 ± 0.61	10.04 ± 0.27	MPR< 2	N/A

^*a*^ Average ± SD.

^*b*^ Not Applicable.

### Bleach is an effective disinfectant for HY-contaminated laboratory surfaces, while 70% EtOH and H_2_O are ineffective disinfectants

**Fig 3:**
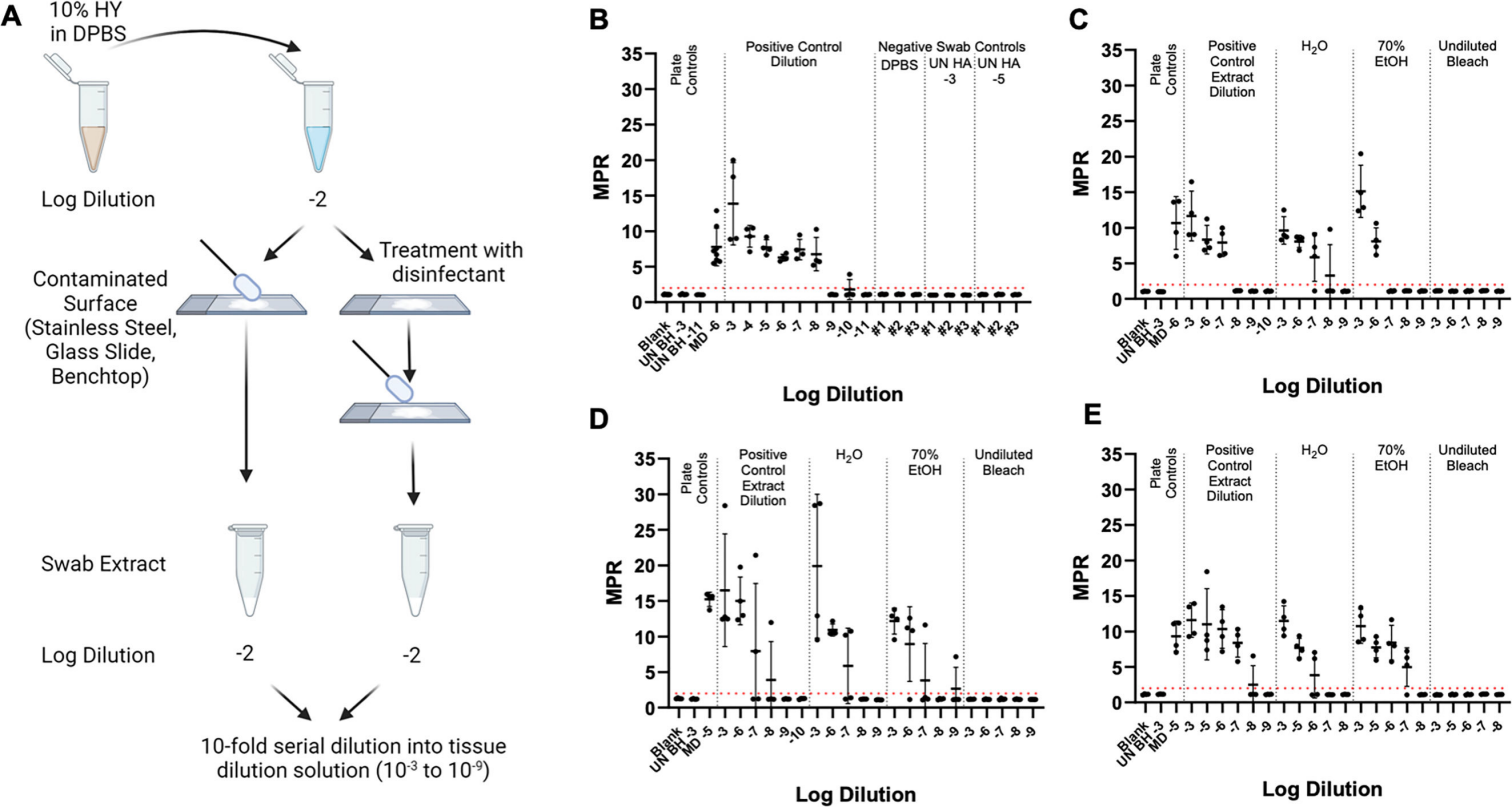
Bleach is an effective disinfectant for HY-contaminated laboratory surfaces, while 70% EtOH and H_2_O are ineffective disinfectants. (**A**)Horizontal dilution method surface contamination and swabbing methodology of contaminated and treated laboratory surfaces (created with BioRender.com). (**B**)RT-QuIC detection for glass slide surface recovered negative controls included UN HA BH 10^−3^, UN HA BH 10^−5^, and DPBS. (**C**)RT-QuIC detection for swab extracts from HY-contaminated glass slide surfaces treated with H_2_O, 70% ethanol, or undiluted bleach for 10 minutes. (**D**)RT-QuIC detection for swab extracts from HY-contaminated stainless steel surfaces treated with H_2_O, 70% ethanol, or undiluted bleach for 10 minutes. (**E**)RT-QuIC detection for swab extracts from HY-contaminated benchtop surfaces treated with H_2_O, 70% ethanol, or undiluted bleach for 10 minutes. Negative plate controls include blank (tissue dilution solution) and uninfected brain homogenate of 10^−3^. A positive plate control consisted of 10^−5^ or 10^−6^ dilution prepared from the mother dilution applied to surfaces. A positive fluorescence threshold (illustrated by the red line) was determined to be at 2. The maxpoint ratio reported is the ratio of the maximum fluorescence to the initial fluorescence reading obtained by the plate reader (mean ± standard deviation). Each experiment was performed at least three times, with similar results obtained from each experiment.

The efficacy of multiple disinfectants in decontaminating prion-contaminated surfaces was investigated. These disinfectants included undiluted bleach and 70% EtOH. Water was used as a negative disinfection control. Each surface type was contaminated with 10^−2^ HY TME-infected brain homogenate, and surfaces were allowed to dry for 24 hours prior to disinfection, swabbing, extraction, and RT-QuIC analysis (Fig. 3, panel A). Each disinfectant was applied to the prion-contaminated area for a treatment duration of 10 minutes. Swab extracts were then evaluated with RT-QuIC, and each plate compared an untreated positive surface control extract to surfaces treated with H_2_O, 70% EtOH, and undiluted bleach. Negative swab controls failed to yield fluorescence signals above the positive fluorescence threshold determined to be at 2 (Fig. 3, panel B). In rare instances, 1/4 technical replicate wells for negative control swab extracts showed low seeding capabilities. The treatment of glass slides, stainless steel surfaces, and benchtops with undiluted bleach led to a complete loss of fluorescence signals above the positive fluorescence threshold of 2 (Fig. 3, panel C). Treatment of glass slide surfaces with both 70% EtOH and H_2_O led to an approximate one log reduction in the SD_50_ when compared to the swab extracts of untreated, prion-contaminated glass slide surfaces (Table 2). Treatment of stainless steel surfaces with H_2_O did not lead to a statistically significant (0 > 0.05) reduction in SD_50_ when compared to the untreated surface. In addition, there was an approximate 0.5 log reduction in SD_50_ for the 70% EtOH-treated stainless steel surfaces, but this difference was not statistically significant (*P* > 0.05). Seventy percent EtOH and H_2_O treatment of benchtop surfaces led to statistically significant (*P* < 0.05) reduction in SD_50_ at approximately 0.7 and 1.3 logs, respectively. Overall, bleach is an effective disinfectant for prion-contaminated surfaces, while H_2_O and 70% EtOH are ineffective.

### Direct surface measurement of prion seeding activity on stainless steel wire mimics the results of surface-recovered prion seeding activity

**Fig 4:**
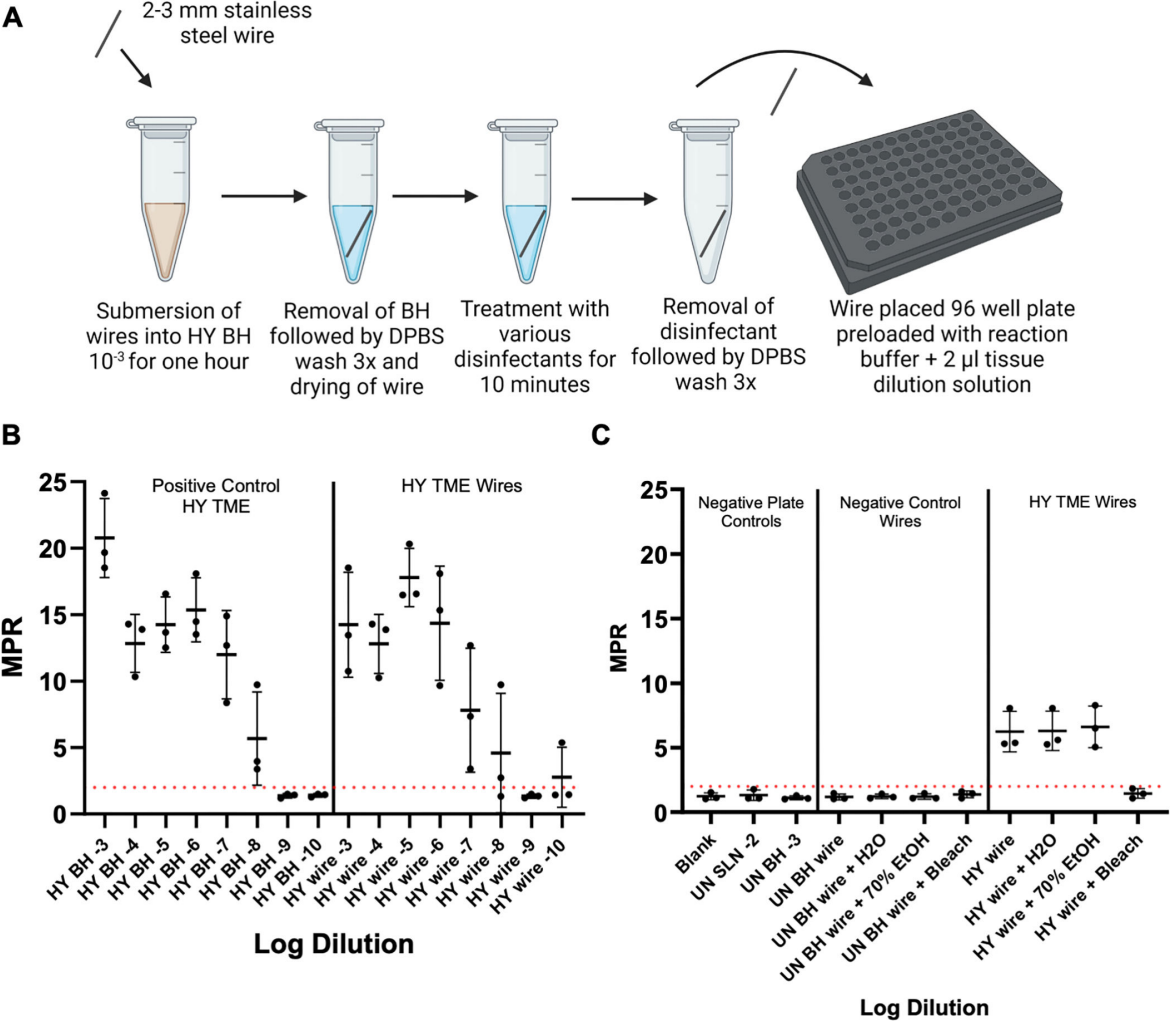
Direct surface measurement of prion seeding activity on stainless steel wire mirrors the results of surface-recovered prion seeding activity. (**A**)Overview of wire contamination and analysis by RT-QuIC (created with BioRender.com). (**B**)RT-QuIC detection of HY BH dilutions and stainless steel wires contaminated with HY BH dilutions. (**C**)RT-QuIC detection of contaminated stainless steel wires. A positive fluorescence threshold (illustrated by red line) was determined to be at 2. The maxpoint ratio reported is the ratio of the maximum fluorescence to the initial fluorescence reading obtained by the plate reader. Each point represents the average MPR from one biological replicate (mean ± standard deviation). For treated HY TME wires, each point is an average of 16 replicate wires, except one biological replicate for 70% EtOH-treated wires contained 15 replicate wires, and one biological replicate of H_2_O-treated wires contained 14 replicate wires.

Mock-treated, plus HY TME-contaminated stainless steel wires were subjected to treatment with either H_2_O, 70% EtOH, or undiluted bleach and were subsequently assayed with RT-QuIC in order to test residual infectivity that may remain on the surface itself following treatment (Fig. 4, panel A). Two- to three-millimeter stainless steel wires were incubated with either UN HA BH 10^−3^ or HY 10^−3^ BH for 1 hour followed by thorough rinsing. Both sets of wires were then subjected to treatment with various disinfectants for 10 minutes prior to rinsing and were then added directly to the 96-well plate for assay with RT-QuIC. The treatment of HY-contaminated stainless steel wires with undiluted bleach led to a complete loss of fluorescence signals above the positive fluorescence threshold of 2 (Fig. 4, panel B). In addition, 70% EtOH and H_2_O were ineffective disinfectants for HY-contaminated stainless steel wires. In addition, negative control wires that underwent the same treatments failed to yield fluorescence signals above the positive fluorescence threshold of 2. All treated HY TME treatment data points are an average of 16 replicate wires, except one biological replicate of 70% EtOH-treated wires, which contained 15 replicate wires, and 1 biological replicate of H_2_O-treated wires, which contained 14 replicate wires.

### Examination of swab extracts by RT-QuIC and animal bioassay for prions

**Fig 5:**
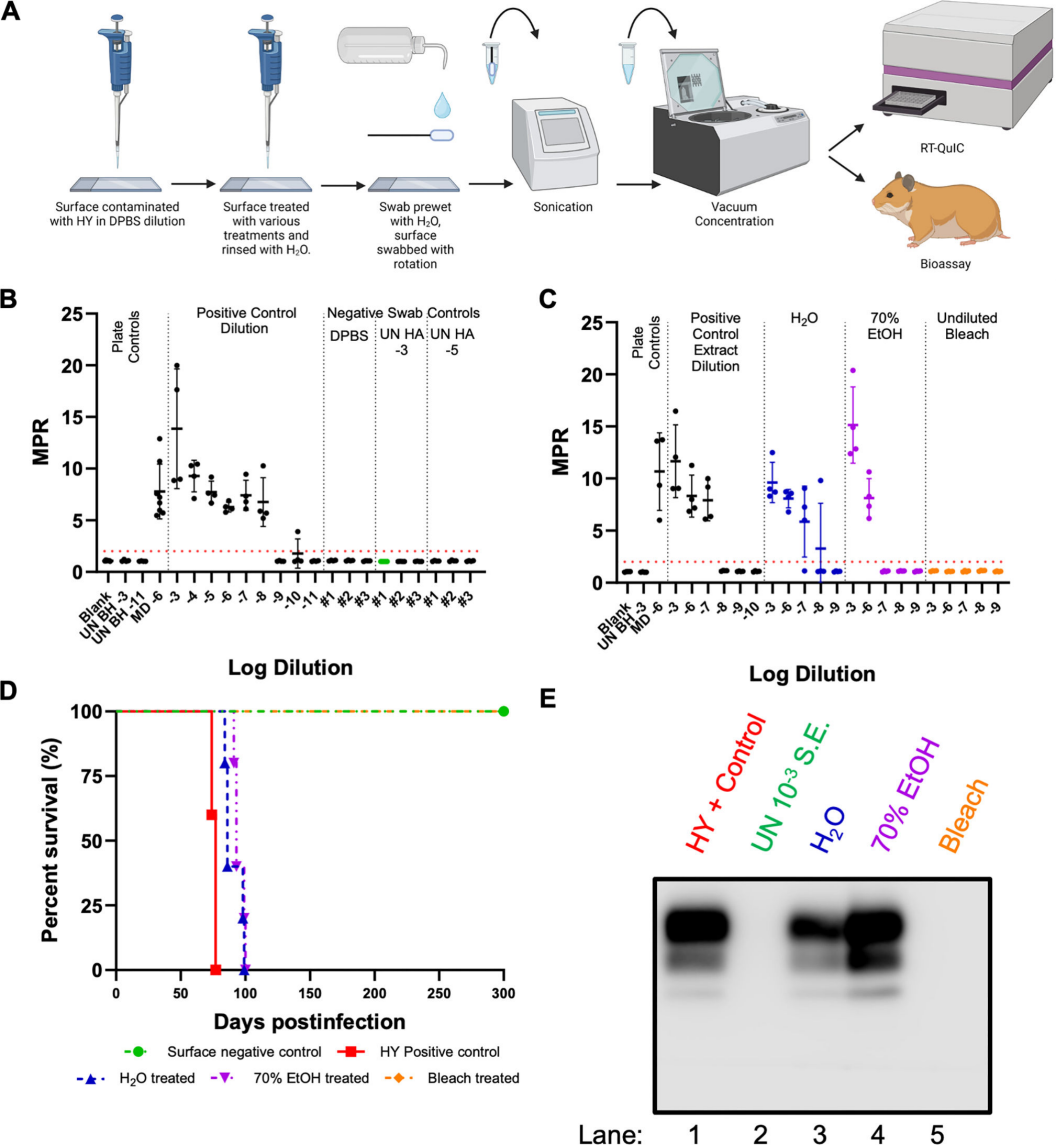
RT-QuIC and animal bioassay of swab extracts produced similar results. (**A**)Overview of surface prion contamination and swabbing procedure resulting in swab extracts that were analyzed by either (**B and C**)RT-QuIC (created with BioRender.com) (**D**)or animal bioassay. A positive fluorescence threshold (illustrated by the red line) was determined to be at 2. The maxpoint ratio reported is the ratio of the maximum fluorescence to the initial fluorescence reading obtained by the plate reader (mean ± standard deviation). (**E**)Western blot analysis of PK digested brain homogenates from bioassay results from panel D.

Mock-contaminated glass and HY TME-contaminated glass treated with either H_2_O, 70% EtOH, or bleach were swabbed, and the swab extract was split into two equal aliquots (Fig. 5, panel A). The first aliquot was analyzed using RT-QuIC for the presence of seeding activity. In the negative control glass surfaces treated with either DPBS, 10^−3^, or 10^−5^ dilution of uninfected (UN) brain homogenate did not result in detectable RT-QuIC seeding activity (Fig. 5, panel B). Swab extracts from glass surfaces contaminated with HY TME that were treated with either H_2_O or 70% EtOH yielded an SD_50_ within approximately 1 log when compared to swab extracts that did not undergo treatment (*P* = 0.09172). Bleach treatment of contaminated glass surfaces resulted in a complete loss of fluorescence above the defined threshold. The second aliquot was intracerebrally (i.c.) inoculated into hamsters to examine for prion infectivity. As a positive control, hamsters were i.c. inoculated with a 10^−4^ dilution of HY TME-infected brain homogenate resulting in all (*n* = 5) of the animals developing clinical signs of hyperexcitability and ataxia at 76 ± 3 days post-infection (dpi) (Fig. 5, panel D). As a negative control, swab extract from an uncontaminated glass surface resulted in none (*n* = 5) of the animals developing clinical signs of prion infection at 300 dpi when the experiment was terminated (Fig. 5, panel D). Swab extracts from HY TME-contaminated surfaces treated with either water or 70% EtOH resulted in all (*n* = 5) of the animals developing clinical signs of prion disease at 91 ± 7 and 95 ± 4 dpi, respectively. Swab extract from HY TME-contaminated surfaces treated with bleach resulted in none (*n* = 5) of the animals developing clinical signs of prion disease at 300 dpi when the experiment was terminated (Fig. 5, panel D). Western blot analysis for the presence of PrP^Sc^ from these animals was consistent with the clinical diagnosis of disease (Fig. 5, panel E). Overall, the results from RT-QuIC and animal bioassay were congruent (Table 3).

**TABLE 3:** Comparison of RT-QuIC and bioassay results of treated prion-contaminated glass slide surfaces

	RT-QuIC SD_50_/g of brain homogenate	RT-QuIC positive wells (10^−3^)	Incubation period (days)^*a*^	Attack rate	PrP^Sc^ present on western blot
UN HA BH swab extract negative control	MPR< 2	0/4	>300	0/5	0/5
HY TME 10^−4^ positive control	11.37	4/4	76 ± 3	5/5	5/5
H_2_O-treated swab extract	10.20	4/4	91 ± 7	5/5	5/5
70% ethanol-treated swab extract	9.20	4/4	95 ± 4	5/5	5/5
Bleach-treated swab extract	MPR< 2	0/4	>300	0/5	0/5

^*a*^ Average ± SD.

### The effect of environmental factors on RT-QuIC reactions

**Fig 6:**
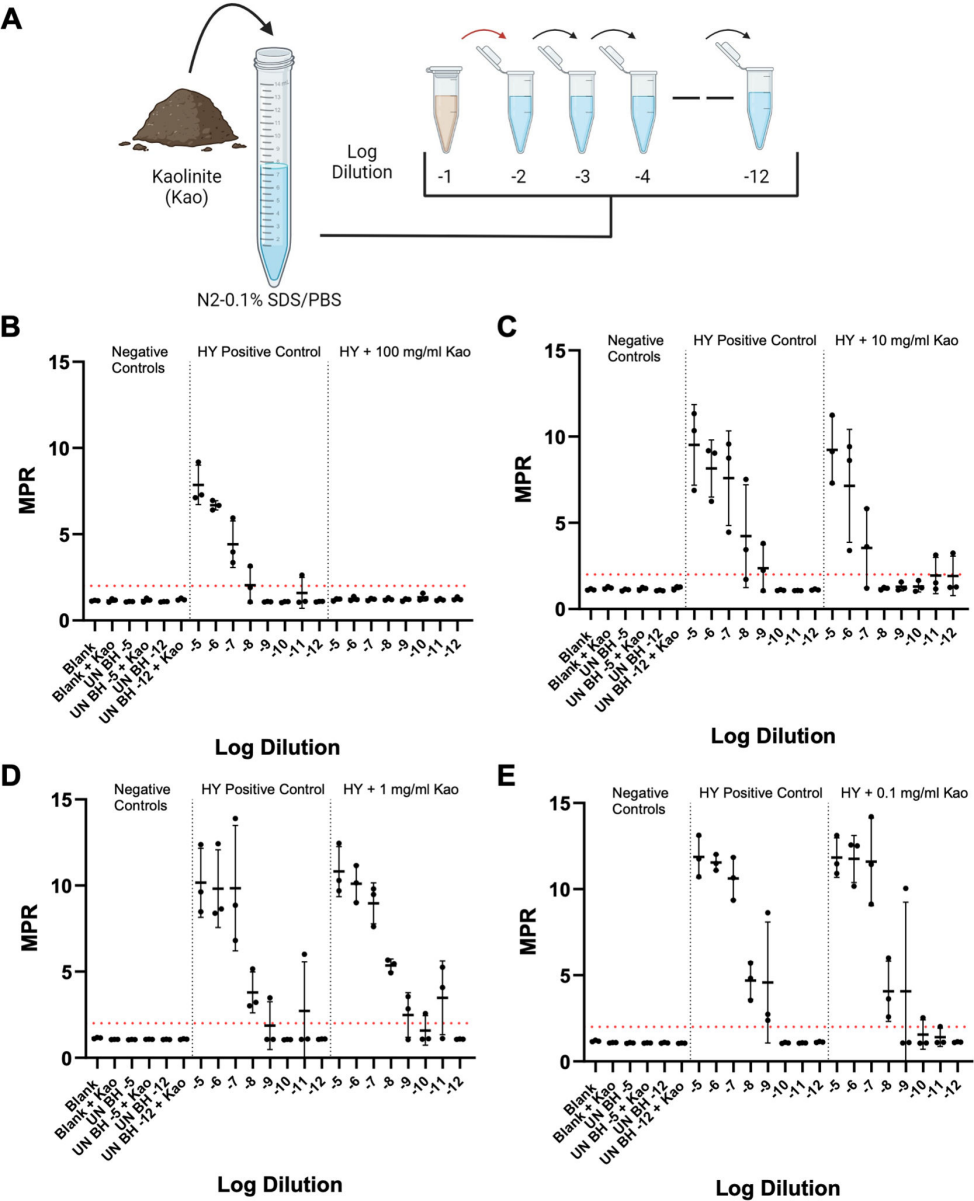
Impact of soil on RT-QuIC detection of HY dilutions is dependent on soil concentration. (**A**)Overview of soil spiking procedure of tissue dilution solution for RT-QuIC analysis (created with BioRender.com). (**B**)RT-QuIC detection of HY dilutions prepared in tissue dilution solution with 100mg/mL of kaolinite. (**C**)RT-QuIC detection of HY dilutions prepared in tissue dilution solution with 10mg/mL of Kao. (**D**)RT-QuIC detection of HY dilutions prepared in tissue dilution solution with 1mg/mL of Kao. (**E**)RT-QuIC detection of HY dilutions prepared in tissue dilution solution with 0.1mg/mL of Kao. Negative plate controls include blank and uninfected brain homogenate of 10^−5^ and 10^−12^ with and without soil. A positive plate control consisted of a HY dilution series prepared in standard tissue dilution solution. A positive fluorescence threshold (illustrated by red line) was determined to be at 2. The maxpoint ratio reported is the ratio of the maximum fluorescence to the initial fluorescence reading obtained by the plate reader. Each point represents the average MPR from one biological replicate (mean ± standard deviation).

Given the wide-ranging use and degree of cleanliness of relevant clinical and laboratory surfaces, various concentrations of three distinct soil minerals [non-expanding kaolinite (Kao), expanding montmorillonite, and hectorite] were added to the tissue dilution solution used for the preparation of UN HA BH negative plate controls and HY TME dilution series to investigate their potential impacts on the RT-QuIC reaction. Each of the aforementioned soil types was added to the tissue dilution solution at concentrations of 100, 10, 1, and 0.1 mg/mL, and this tissue dilution solution was used for the preparation of UN HA BH and HY TME dilution series (Fig. 6, panel A). The addition of the highest concentration of Kao led to a complete inhibition of the detection of the HY TME dilution series, yielding no fluorescence above the set positive fluorescence threshold of 2 (Fig. 6, panel B). Interestingly, the fluorescence curves of the uninfected BH dilutions with 100 mg/mL of Kao added showed distinct tracings (although below the positive threshold) when compared to the same dilutions without soil added (Fig. S7, panel A). The addition of 10, 1, and 0.1 mg/mL did not significantly (*P* < 0.05) alter the SD_50_ of the HY dilution series compared to a no soil control (Fig. 5, panels C, D, and E). For hectorite, the addition of 10 mg/mL completely inhibited the RT-QuIC reaction, while the addition of 1 and 0.1 mg/mL did not significantly (*P* < 0.05) affect the SD_50_ value sensitivity (Table 4). For montmorillonite (Mte), again the addition of 10 mg/mL of soil inhibited the RT-QuIC reaction. While 0.1 mg/mL of Mte had no significant (*P* > 0.05) effect on the sensitivity of the RT-QuIC reaction, the addition of 1 mg/mL led to an approximate 1 log increase in SD_50_, which was statistically (*P* < 0.05) significant. In addition, 0.1 mg/mL Mte led to a decrease in the maximum fluorescence observed in each replicate well (Fig. S7, panel B). Of note, when performing experiments on factory new benchtop surfaces, an altered background fluorescence curve was observed, extending above the positive fluorescence threshold (Fig. S7, panel C), which was eliminated by washing with water (Fig. S7, panel C). Due to these findings, the impact of environmental contaminants such as dust and byproducts of manufacturing on RT-QuIC should be considered.

**TABLE 4:** Comparison of log SD_50_/g of brain homogenate, determined by RT-QuIC end-point dilutions, of the effects of soil on HY dilution seeding capabilities

Mineral	Soil concn (mg/mL)	HY	1	2	3	Avg± SD
Kaolinite		HY without soil	9.93	10.03	10.90	10.29 ± 0.44
	100	HY with soil	MPR< 2	MPR< 2	MPR< 2	MPR< 2
		HY without soil	10.20	11.70	10.93	10.94 ± 0.61
	10	HY with soil	8.37	10.31	9.96	9.55 ± 0.84
		HY without soil	10.70	12.25	10.37	11.11 ± 0.82
	1	HY with soil	11.52	12.25	10.70	11.49 ± 0.63
		HY without soil	10.70	10.93	10.93	10.85 ± 0.11
	0.1	HY with soil	11.82	10.37	11.31	11.17 ± 0.60
Montmorillonite		HY without soil	10.70	10.03	10.20	10.31 ± 0.28
	10	HY with soil	MPR< 2	MPR< 2	MPR< 2	MPR< 2
		HY without soil	10.20	12.08	10.47	10.92 ± 0.83
	1	HY with soil	11.70	11.70	12.14	11.85 ± 0.21^*a*^
		HY without soil	10.47	11.03	10.03	10.51 ± 0.41
	0.1	HY with soil	11.70	11.47	10.20	11.12 ± 0.66
Hectorite		HY without soil	9.92	10.37	9.37	9.89 ± 0.41
	10	HY with soil	MPR< 2	MPR< 2	MPR< 2	MPR< 2
		HY without soil	9.37	10.03	10.46	9.95 ± 0.45
	1	HY with soil	9.93	10.20	11.03	10.39 ± 0.47
		HY without soil	10.47	10.03	10.70	10.40 ± 0.28
	0.1	HY with soil	10.20	10.70	11.44	10.78 ± 0.51

^*a*^ p<0.05.

### The addition of nanoparticles to RT-QuIC can increase the sensitivity of surface-recovered prions

**Fig 7:**
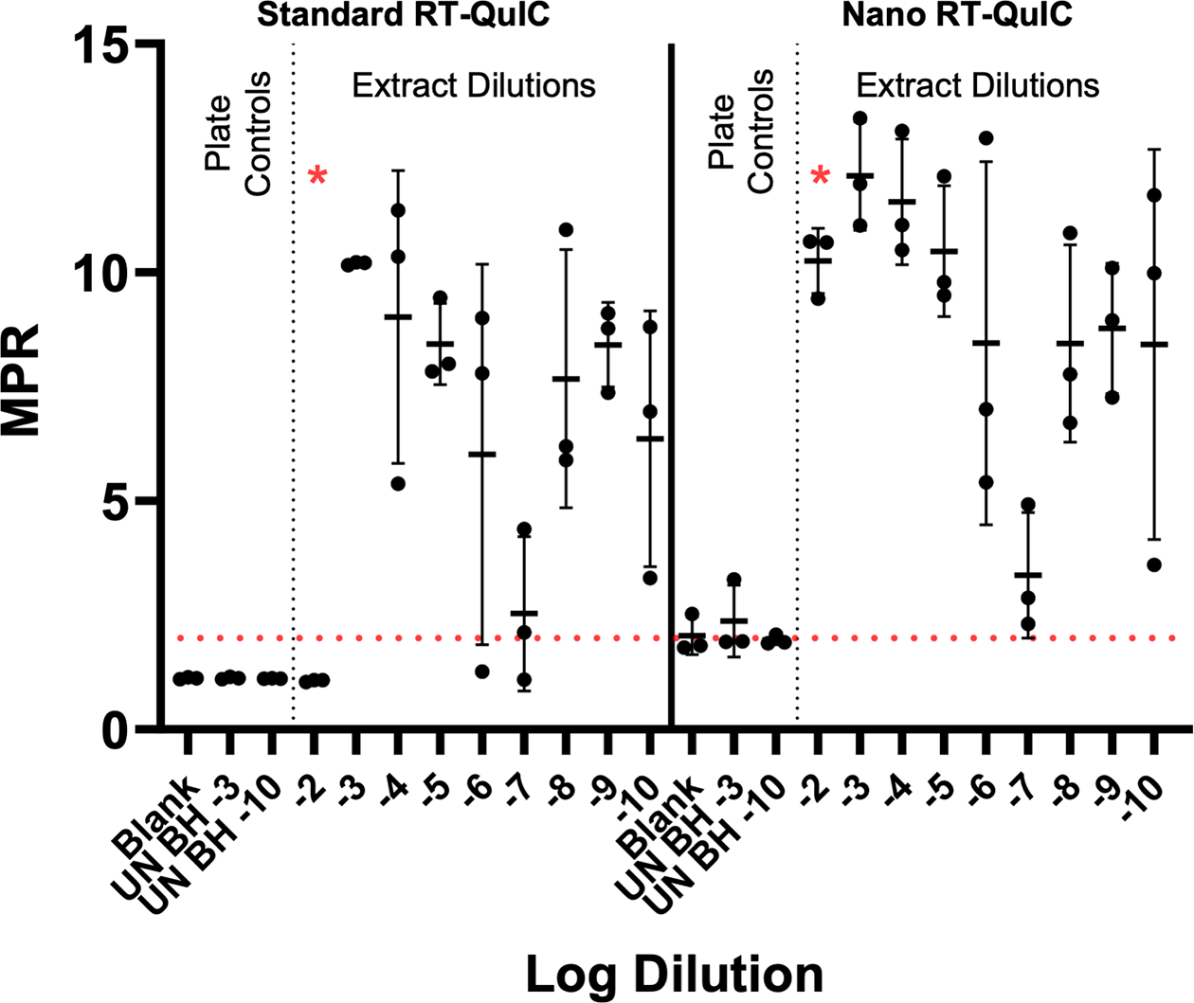
Nano RT-QuIC increases the sensitivity of RT-QuIC detection of surface-recovered HY. RT-QuIC and nano RT-QuIC detection of stainless steel surface-recovered HY dilutions from vertical dilution experiment. Negative plate controls include blank and uninfected brain homogenate dilution of 10^−3^ and 10^−10^. A positive fluorescence threshold (illustrated by the red line) was determined to be at an MPR of 2. The maxpoint ratio reported is the ratio of the maximum fluorescence to the initial fluorescence reading obtained by the plate reader. Each point represents the average MPR from one technical replicate (mean ± standard deviation).

The ability of silica nanoparticles to increase the sensitivity of RT-QuIC on surface-recovered HY prions was investigated. Stainless steel tokens were contaminated with 10^−1^ to 10^−9^ 10-fold serial dilutions of HY TME-infected brain homogenate in DPBS (mother dilutions), and contaminated surfaces were allowed to dry for 24 hours prior to swabbing, extraction, and RT-QuIC analysis as previously described. While low dilutions of brain homogenate are not generally tested due to the inhibitory effects on RT-QuIC, the addition of silica nanoparticles has previously been shown to overcome this inhibition (98). For this reason, a dilution series of 10^−2^ to 10^−10^ was investigated using both standard RT-QuIC and nanoparticle RT-QuIC (Nano-QuIC). Negative plate controls for standard RT-QuIC failed to yield MPR above the determined positive threshold of 2 (Fig. 7). One out of three replicates yielded positive signals in the negative plate controls for nanoparticle RT-QuIC reactions. When comparing standard RT-QuIC to nanoparticle RT-QuIC, a restoration of seeding was observed for the HY dilution 10^−2^ following the addition of nanoparticles, thus increasing the sensitivity of surface recovered HY prions by one order of magnitude.

## DISCUSSION

The surface swab prion detection method effectively assessed the decontamination efficacy of two common disinfectants. Building upon our previous work indicating that prions can be recovered from surfaces with swabbing, here we show efficient recovery of prions from laboratory and clinically relevant surfaces (Fig. 1) (97). To investigate the utility of this methodology in assessing the effectiveness of prion decontamination procedures, we treated surfaces with either water as a negative decontamination control, 70% EtOH as an example of a common laboratory disinfectant, and sodium hypochlorite as a positive control for prion decontamination. We observed that 70% EtOH treatment of HY TME-contaminated surfaces resulted in the detection of RT-QuIC seeding activity in the swab extracts with a similar SD_50_ compared to swab extracts from water-treated HY TME-contaminated surfaces (Fig. 3) consistent with previous studies suggesting the ineffectiveness of ethanol as a prion disinfectant (39, 42, 99, 100). Consistent with previous findings, RT-QuIC testing of swab extracts from bleach-treated surfaces failed to detect prion RT-QuIC seeding activity (Fig. 3) (58, 86, 89, 101). Importantly, control experiments determined that the failure to detect prions on bleach-treated surfaces was not due to residual bleach in the surface swab extract inhibiting the RT-QuIC reaction but was instead due to the destruction of prions (Fig. 2).

The reduction in RT-QuIC seeding activity of surface swab extracts corresponded with a reduction in the seeding activity of prion-coated stainless steel wires. To investigate the possibility that the reduction in RT-QuIC seeding activity of the surface swab extracts is due to a failure to recover infectious prions from the surface, we coated stainless steel wires with HY TME brain homogenate and placed them directly into RT-QuIC reaction tubes to test for prions bound to the surface (58). As a positive control, stainless steel wires were coated with a 10-fold dilution series of HY TME-infected brain homogenate and placed directly into the RT-QuIC reactions, resulting in a similar sensitivity for prion detection as HY TME homogenate directly added to the RT-QuIC reaction (Fig. 4, panel B). Treatment of the HY TME-contaminated stainless steel wires with either water or 70% EtOH resulted in similar RT-QuIC seeding activity consistent with a lack of prion inactivation observed in the surface swab extracts (Fig. 4, panel C). Treatment of the HY TME-contaminated prion wires with bleach resulted in a failure to detect RT-QuIC seeding activity on the wire surface (Fig. 4, panel C) similar to previous studies (58, 86). The combination of the failure to detect RT-QuIC seeding activity from surface swab extracts and bleach-treated stainless steel wires indicates that the surface swab extract findings are predictive of the prion contamination status of the surface tested.

The RT-QuIC seeding activity and prion infectivity of surface swab extracts are congruent. To investigate the possibility that RT-QuIC seeding activity may not correspond with prion infectivity, we designed an experiment where the same surface extracts were tested for seeding activity using RT-QuIC and i.c. inoculated into hamsters to examine for prion infectivity. We found that surfaces treated with either water or 70% EtOH contained RT-QuIC seeding activity, which also resulted in all the animals in each group developing prion disease (Fig. 5). Interestingly, the seeding activity SD_50_ and incubation period in hamsters were similar between the water- and 70% EtOH-treated surface swab extracts, suggesting that both methods produced qualitatively similar results (Fig. 5) (102). The bleach-treated surface swab extracts failed to result in RT-QuIC seeding activity and did not result in clinical signs of prion disease in hamsters or evidence of a subclinical infection, at 300 days post-infection (Fig. 5). As 300 days post-infection is a longer incubation period than a single i.c. LD_50_ of HY TME, we interpret this finding that bleach treatment completely inactivated HY TME infectivity (103, 104). As we applied 5 × 10^5.8^ i.c. LD_50_ of prion infectivity to the surface, we interpret this negative result as a >5-log reduction in prion infectivity. Overall, under the conditions tested, we found that RT-QuIC seeding activity and animal bioassay produced similar results.

Nano-QuIC may enhance the sensitivity of prion detection from surface swab extracts. A weakness of the current study is that the swab extract must be diluted 100-fold prior to RT-QuIC analysis, as higher concentrations (i.e., lower dilutions) of swab extract inhibit the detection of PrP^Sc^ in RT-QuIC (Fig. 7). This inhibitory phenomenon has previously been observed with low dilution of tissue homogenates (102, 105–107). A recently described modification of RT-QuIC that includes nanoparticles (nano-QuIC) may overcome this limitation (98). Here, we found that the use of nano RT-QuIC resulted in the reliable detection of prions in 10-fold swab extract dilutions, effectively increasing the sensitivity of the assay by an order of magnitude (Fig. 7). While promising, the effectiveness of Nano-QuIC to overcome RT-QuIC inhibitors may differ with the nature of the contents of the extract or the prion strain tested. Further studies are warranted to evaluate the effectiveness of nano RT-QuIC for surface swab extract detection of prions.

Testing surfaces for residual prion infectivity can be integrated into a comprehensive prion safety program. A recent report provided a methodology to test surfaces and tools to verify that prion decontamination was effective (96). Here, we extended this important work by integrating surface swabbing that may prove to be useful where the application of media for direct testing by RT-QuIC is impractical. In laboratory settings, this can be used to verify that laboratory space is decontaminated and to evaluate the potential for cross-contamination from disinfected necropsy tools used between animals and/or tissues. Similarly, in clinical settings, surfaces and instruments can be tested for the presence of residual prions to reduce the risk of iatrogenic transmission. Here, we show the limitations of the technique as the addition of soil and residues from the manufacturing process of laboratory benchtop can adversely affect RT-QuIC results (Fig. 6; Fig. S7; Table 4). From a practical perspective, laboratory and clinical surfaces should be as free of dust and chemicals prior to surveying for prion seeding activity. Anomalous RT-QuIC results from surfaces not described here should be interpreted with caution. Additional analysis or experimentation to discriminate between bona fide seeding activity and environmental artifact would be justified. Finally, the methodologies described here can be adapted for prion and prion-like diseases that support detection using RT-QuIC (92–94, 96, 108, 109).

## MATERIALS AND METHODS

### Prion sources

Brain tissue was collected from hamsters infected with hyper transmissible mink encephalopathy at the terminal stage of disease containing 10^9.3^ i.c. LD_50_/g of prion infectivity as previously described (104). Brain tissue was homogenized in Dulbecco’s phosphate-buffered saline (Mediatech, Herndon, VA, USA) to 10%, wt/vol using syringe and needle homogenization. The 10%, wt/vol HY BH aliquots were clarified by centrifugation at 450 *g* for 30 seconds. Supernatants were transferred into new 1.5 mL microcentrifuge tubes and stored at −80°C.

### Surface contamination—vertical dilution experiments

Ten percent uninfected brain homogenate (BH) was used to prepare serial 10-fold dilutions in DPBS (10^−2^–10^−9^) for use as negative controls. The dilutions applied directly to the surface will be referred to as mother dilutions. Ten percent HY BH was used to prepare serial 10-fold mother dilutions in DPBS (10^−2^–10^−9^). Fifty microliters of each dilution in the series was applied in triplicate onto the surface of Sakura, Tissue-Tek SmartWrite Charge, white frosted glass slides (product number: 9036), stainless steel tokens (Lindstrom Grade 304, Part# FW5X01000, Lot# W033000812), or laboratory benchtop surfaces (Kewaunee, Kemresin epoxy resin benchtop, 1″ cubes with one finished surface). Mother dilutions were stored at −80°C for further use in RT-QuIC plates. Glass slide surfaces were partitioned using a hydrophobic marker to prevent the runoff water generated during rinsing from reaching other prion-contaminated areas. Negative controls applied to all surface materials included DPBS, UN 10^−2^, and UN 10^−9^. Fifty microliters of each negative control was applied in triplicate. All surfaces were allowed to dry overnight.

### Surface contamination—horizontal dilution experiment

HY 10% BH was used to prepare 10-fold serial dilutions in DPBS (10^−2^ and 10^−4^ were used for this experiment). UN 10% BH was used to prepare 10-fold serial dilutions in DPBS (10^−2^ and 10^−4^ were used for this experiment). Negative controls applied to all surfaces included DPBS, UN 10^−2^, and UN 10^−4^ BH. Fifty microliters of each mother dilution was applied in triplicate to each respective surface. The remaining mother dilutions were stored at −80°C. All surfaces were allowed to dry overnight.

### Surface disinfection

For surface disinfection, either MQ H_2_O, 70% EtOH, or undiluted bleach (Clorox, Sodium Hypochlorite 8.25%) was used as a treatment. Prior to 70% EtOH treatment, boxes were drawn around the prion-contaminated area on glass slide surfaces with a hydrophobic marker to prevent the dispersal of the treatment. Contaminated glass slide surfaces, stainless steel tokens, and benchtop surfaces were subjected to 70, 60, or 90 µL, respectively, of one of the three disinfection treatments at room temperature for 10 minutes. After 10 minutes, the treated surfaces were rinsed thoroughly with water prior to swabbing. Following rinsing, if the surfaces remained wet, foam swabs were not prewet prior to swabbing, if surfaces were dry, foam swabs were prewet. All treatments were performed in triplicate on separate prion-contaminated areas.

### Surface swabbing, swab extraction, and concentration

Extracts from swabbed surfaces were performed as previously described (97). Briefly, foam swabs (Fisher brand, Catalog #14-960-3E) were prewet with MQ water. Each surface was swabbed with a separate, new swab approximately 10 times with rotation. Swab handles were cut to fit into microcentrifuge tubes preloaded with 250 µL DPBS, and swabs were immediately placed into designated tubes. The swabs, in microcentrifuge tubes, were sonicated (Q700 QSonica sonicator) for a total of 15 seconds (5 seconds of sonication and 5 seconds of rest) at an average of 80 watts. After sonication, swabs were briefly centrifuged (Thermo Scientific mini-centrifuge) prior to the transfer of the extract into a new tube. The swab extracts were then subjected to vacuum concentration (Thermo Scientific savant speedvac SPD1030) for a total of 2 hours at 45°C and a vacuum setting of 5.1. Pellets were resuspended with 50 µL of H_2_O prior to RT-QuIC analysis.

### Bleach RT-QuIC inhibition control experiment

Two groups of glass slides were contaminated with 10^−2^ HY as described above. One prion-contaminated surface was treated with undiluted bleach as described in Surface Disinfection section. The treated and rinsed surface was then swabbed as described above to capture any residual bleach from the surface. This same swab was then used to immediately swab the second 10^−2^ HY-contaminated surface. The swabs in microcentrifuge tubes were then subjected to the swab extraction and concentration protocol described below.

### Dilution preparation for RT-QuIC

For the vertical dilution experiment, each swab extract in a dilution series was 10-fold diluted into N2-0.1% SDS/PBS (N-2 supplement (100×); ThermoFisher catalog #17502048). Positive plate control dilutions for vertical dilution experiments included the original mother dilution series diluted 10-fold into N2-0.1%SDS/PBS (Fig. S1). HY was diluted 10-fold into N2-0.1%SDS/PBS for use as a positive control in swab control plates. For the horizontal dilution experiment, both untreated and treated 10^−2^ swab extracts were subjected to further 10-fold serial dilution in N2-0.1%SDS/PBS (10^−3^–10^−9^). The untreated swab extracts were used as a positive plate control.

### RT-QuIC reaction

RT-QuIC was performed as previously described (97). Briefly, negative plate controls included a blank sample (N2-0.1%SDS/PBS) and the lowest dilution included in the plate of UN BH. The reaction buffer was prepared to the following concentrations: 1× PBS, 170 mM, 1 mM EDTA, 10 µM ThT, and 0.1 mg/mL of recombinant hamster prion protein (recHaPrP; Priogen Corp, St. Paul, MN, USA). Two microliters of each sample was added to 98 µL of the reaction buffer. Cycle duration was 45 minutes, and fluorescence readings were recorded once per cycle for a total of 65 cycles. RT-QuIC reactions were performed in a BMG FLUOstar Omega plate reader (BMG Labtech, Cary, NC, USA). Shaking at 700 rpm double orbital with alternating cycles of 1 minute of shaking followed by 1 minute of rest. The MPR was obtained by dividing the maximum fluorescence reading in each well by the initial (background) fluorescence. A positive fluorescence threshold was established at 2. A cutoff of 45 cycles (33 hours) was established for positive time to fluorescence following the *post hoc* analysis of the time to fluorescence for negative and positive controls from 84 plates. SD_50_ determinations (dilution at which 50% of replicate wells give off fluorescence above the defined positive threshold) were calculated using the method of Reed and Muench (110)

### Nano-QuIC reaction comparison

For Nano-QuIC, experiments were performed as previously described (98). Briefly, RT-QuIC reaction plates were performed with standard RT-QuIC and Nano-QuIC ran in parallel. The Nano-QuIC reaction buffer was prepared to the following concentrations: 1× PBS, 300 mM NaCl, 500 µM EDTA, 50 µM ThT, 2.5 mg/mL 50 nm silica nanoparticles, and 0.1 mg/mL of recombinant hamster prion protein. Negative plate controls included tissue dilution solution, UN HA BH 10^−3^ and UN HA BH 10^−10^. All test samples were investigated with standard reaction buffer and Nano-QuIC reaction buffer within the same plate. RT-QuIC was performed as previously described.

### Wire-QuIC reaction

Wire-QuIC reactions were performed as previously described (58). Briefly, 2–3 mm stainless steel wires were incubated with 50 µL of HY BH 10^−3^, prepared in DPBS, following a brief vortex in microcentrifuge tubes for 1 hour. Following incubation, BH was discarded, and wires were washed with DPBS three times. Wires in microcentrifuge tubes were allowed to dry overnight. For each treatment group, 16 wires were used. A volume of 50 µL of each disinfectant was added to the wires for 10 minutes followed by thorough rinsing. Negative plate controls included a blank sample, UN SLN 10^−2^, and UN HA BH 10^−3^. Negative wire controls included wires incubated with UN HA BH 10^−3^ that underwent either no treatment, treatment with H_2_O, treatment with 70% EtOH, or treatment with undiluted bleach. One wire was added to each well. Each well contained 98 µL of reaction buffer and 2 µL of tissue dilution solution. RT-QuIC was performed as previously described.

### Effects of soil on RT-QuIC seeding capabilities experiment

Briefly, a stock of tissue dilution solution was created for each soil mineral and each soil final concentration. These included kaolinite (100, 10, 1, and 0.1 mg/mL), montmorillonite (10, 1, and 0.1 mg/mL), and hectorite (10, 1, and 0.1 mg/mL). Stocks were generated by adding the appropriate amount of soil to N2-0.1% SDS/PBS followed by vortexing to achieve thorough mixing. A 10-fold dilution series was generated for HY TME BH and UN HA BH in standard N2-0.1%SDS/PBS and soil N2-0.1%SDS/PBS. RT-QuIC was performed as previously described. Negative plate controls included a blank sample (without soil), UN HA BH 10^−5^ (without soil), and UN HA BH 10^−12^ (without soil). Negative soil controls included a blank (with soil added), UN HA BH 10^−5^ (with soil added), and UN HA BH 10^−12^ (with soil added).

### Bioassay

Male 3–4-week-old Syrian hamsters were intracerebrally inoculated with 20 µL of either a 1:10 dilution of swab extracts or a HY 10^−4^ BH dilution. The animals were monitored three times a week for the onset of neurological signs, and the incubation period was calculated as the difference in days between the date of inoculation and the onset of clinical signs of prion infection.

## SUPPLEMENTAL MATERIAL

Figure S1 msphere.00504-24-s0001.tiff Dilution preparation.

Figure S2 msphere.00504-24-s0002.tiff Bleach is an effective disinfectant for HY contaminated stainless steel surfaces.

Figure S3 msphere.00504-24-s0003.tiff Bleach is an effective disinfectant for HY contaminated benchtop surfaces.

Figure S4 msphere.00504-24-s0004.tiff Bleach is an effective disinfectant for HY contaminated glass surfaces.

Figure S5 msphere.00504-24-s0005.tiff Impact of montmorillonite on RT-QuIC detection of HY dilutions is dose dependent.

Figure S6 msphere.00504-24-s0006.tiff Impact of Hectorite on RT-QuIC detection of HY dilutions is dose dependent.

Figure S7 msphere.00504-24-s0007.tiff Addition of environmental contamination impacts RT-QuIC.

Legends msphere.00504-24-s0008.docx Supplemental figure legends.

ASM does not own the copyrights to Supplemental Material that may be linked to, or accessed through, an article. The authors have granted ASM a non-exclusive, world-wide license to publish the Supplemental Material files. Please contact the corresponding author directly for reuse.
