# Supplementary figures and images for: Rapid and sensitive determination of residual prion infectivity from prion-decontaminated surfaces
Source: mSphere. 2024 Aug 27;9(9):e00504-24. doi: 10.1128/msphere.00504-24 (PMC11423590; doi:10.1128/msphere.00504-24)

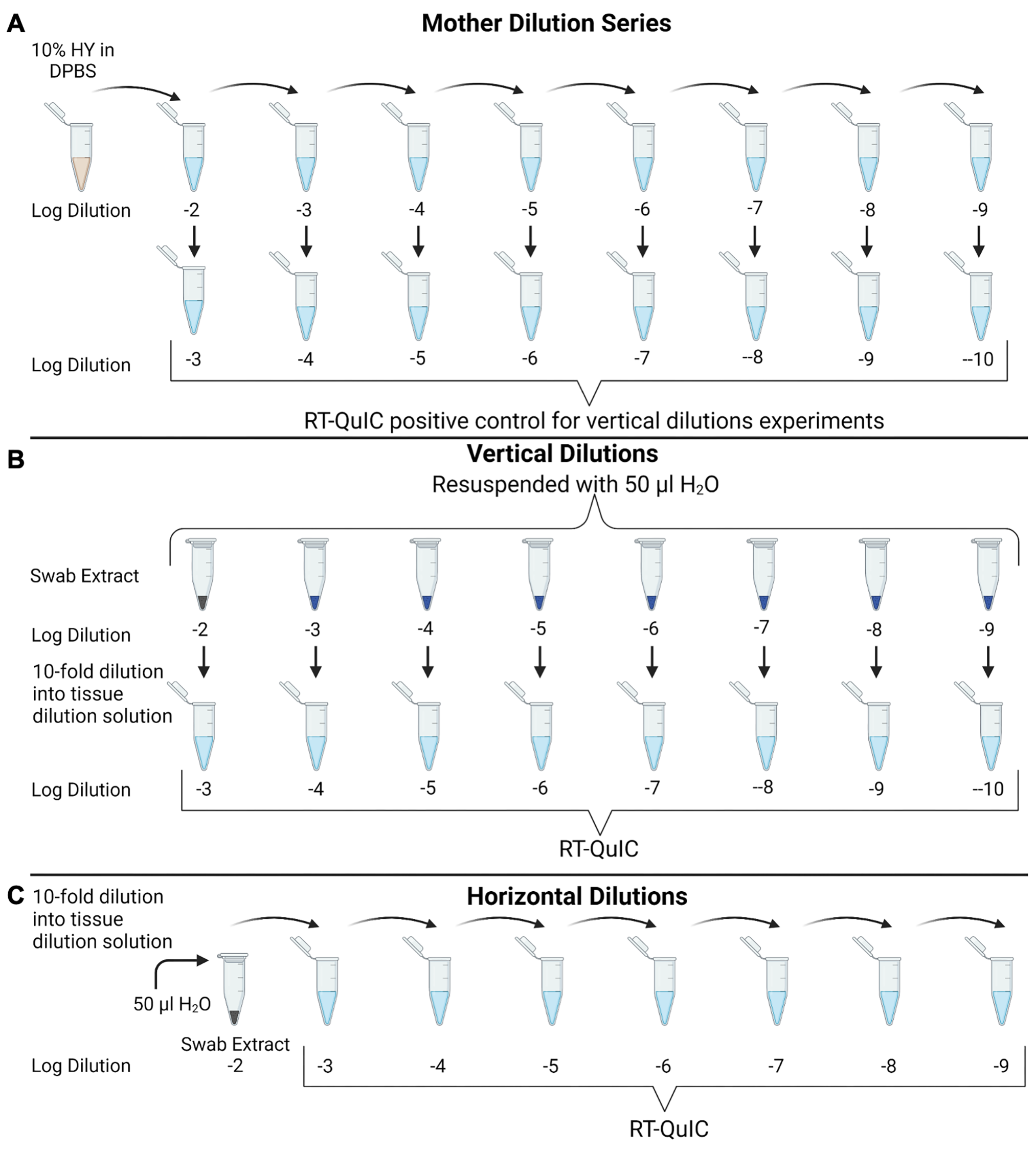

Supplement: Figure S1 — Dilution preparation. [file msphere.00504-24-s0001.tiff]

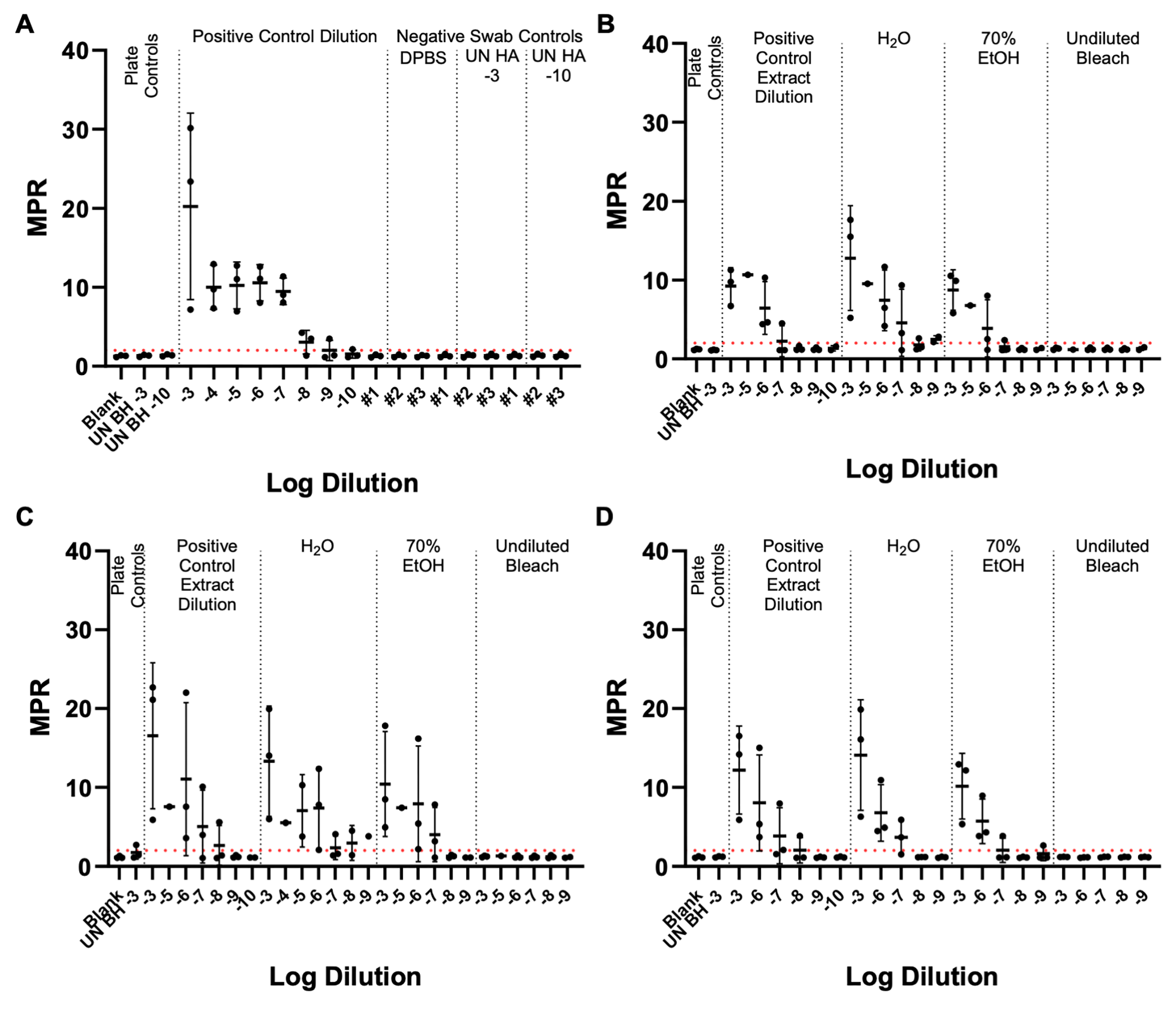

Supplement: Figure S2 — Bleach is an effective disinfectant for HY contaminated stainless steel surfaces. [file msphere.00504-24-s0002.tiff]

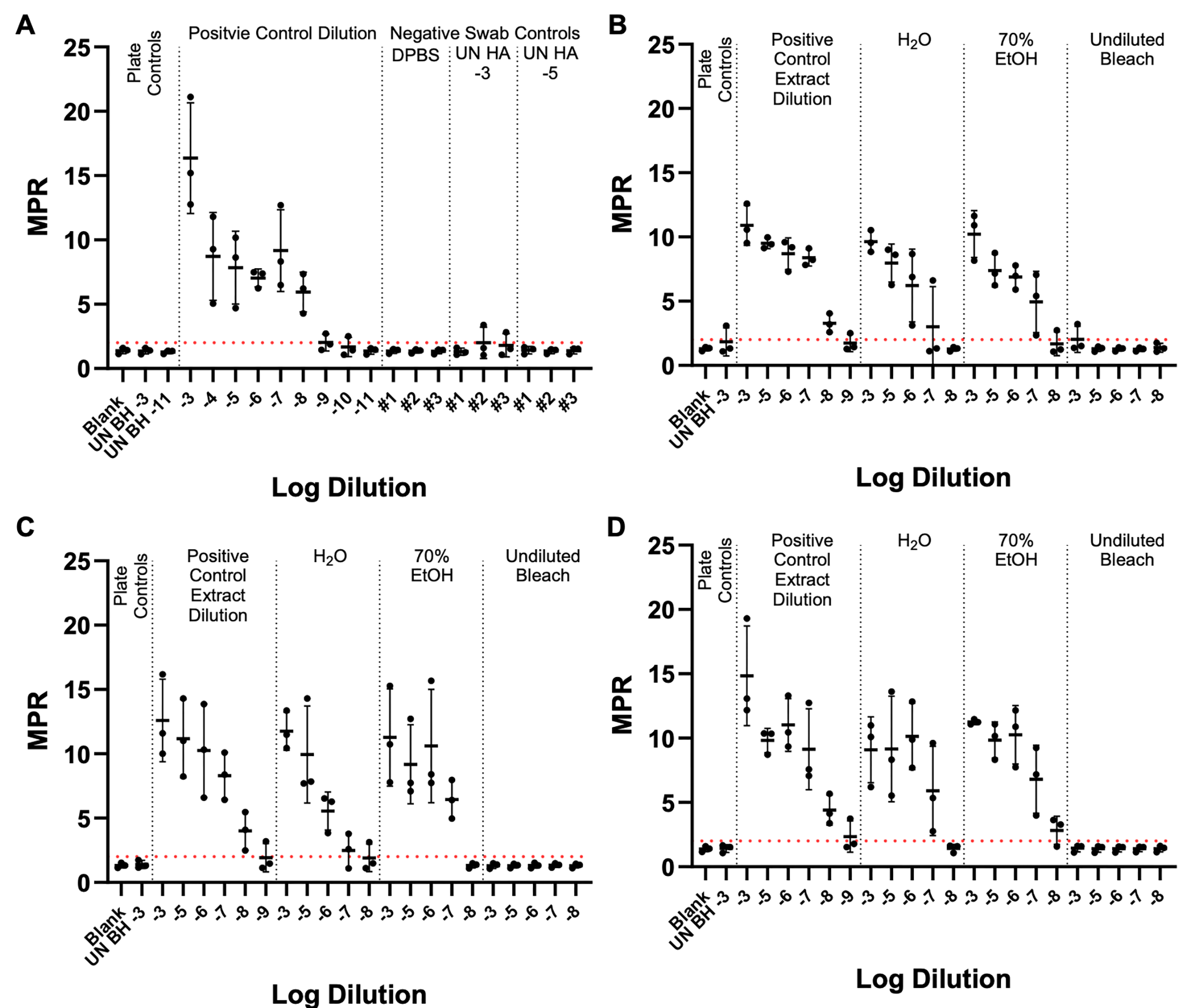

Supplement: Figure S3 — Bleach is an effective disinfectant for HY contaminated benchtop surfaces. [file msphere.00504-24-s0003.tiff]

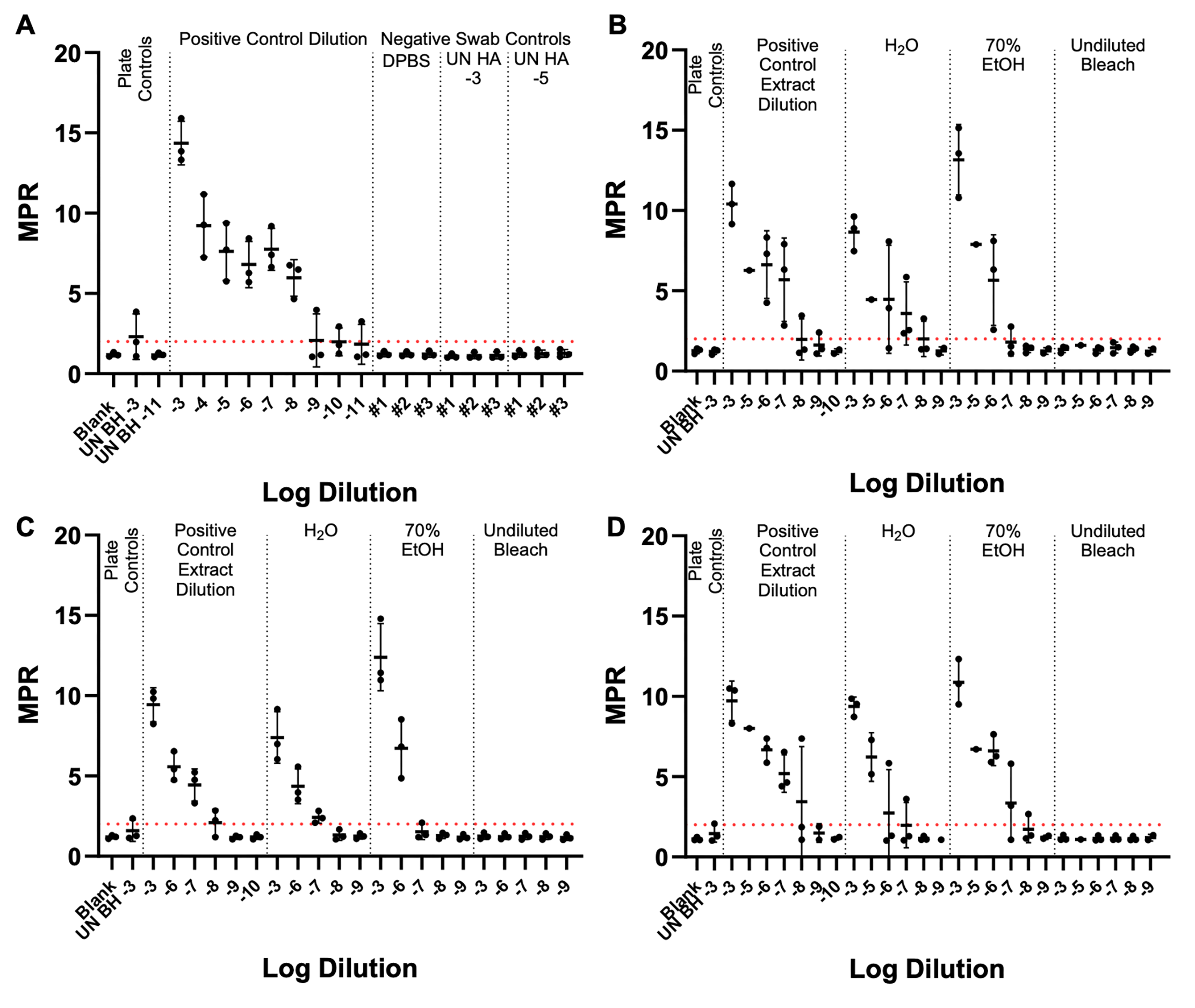

Supplement: Figure S4 — Bleach is an effective disinfectant for HY contaminated glass surfaces. [file msphere.00504-24-s0004.tiff]

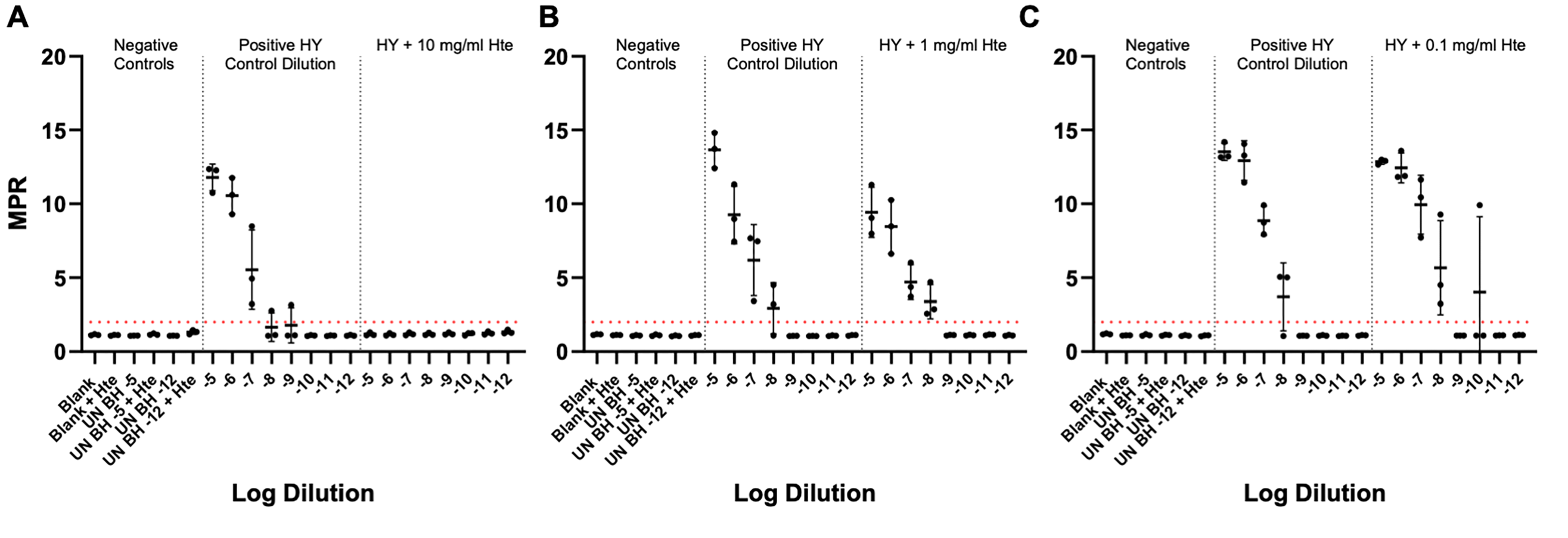

Supplement: Figure S5 — Impact of montmorillonite on RT-QuIC detection of HY dilutions is dose dependent. [file msphere.00504-24-s0005.tiff]

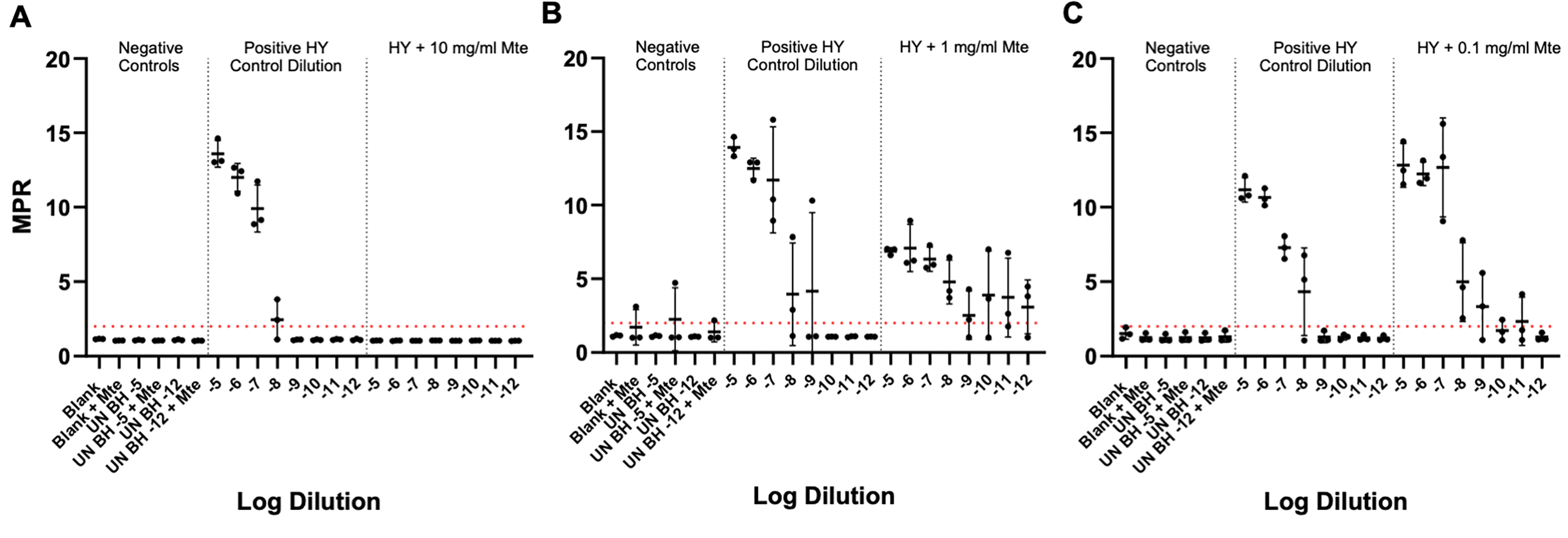

Supplement: Figure S6 — Impact of Hectorite on RT-QuIC detection of HY dilutions is dose dependent. [file msphere.00504-24-s0006.tiff]

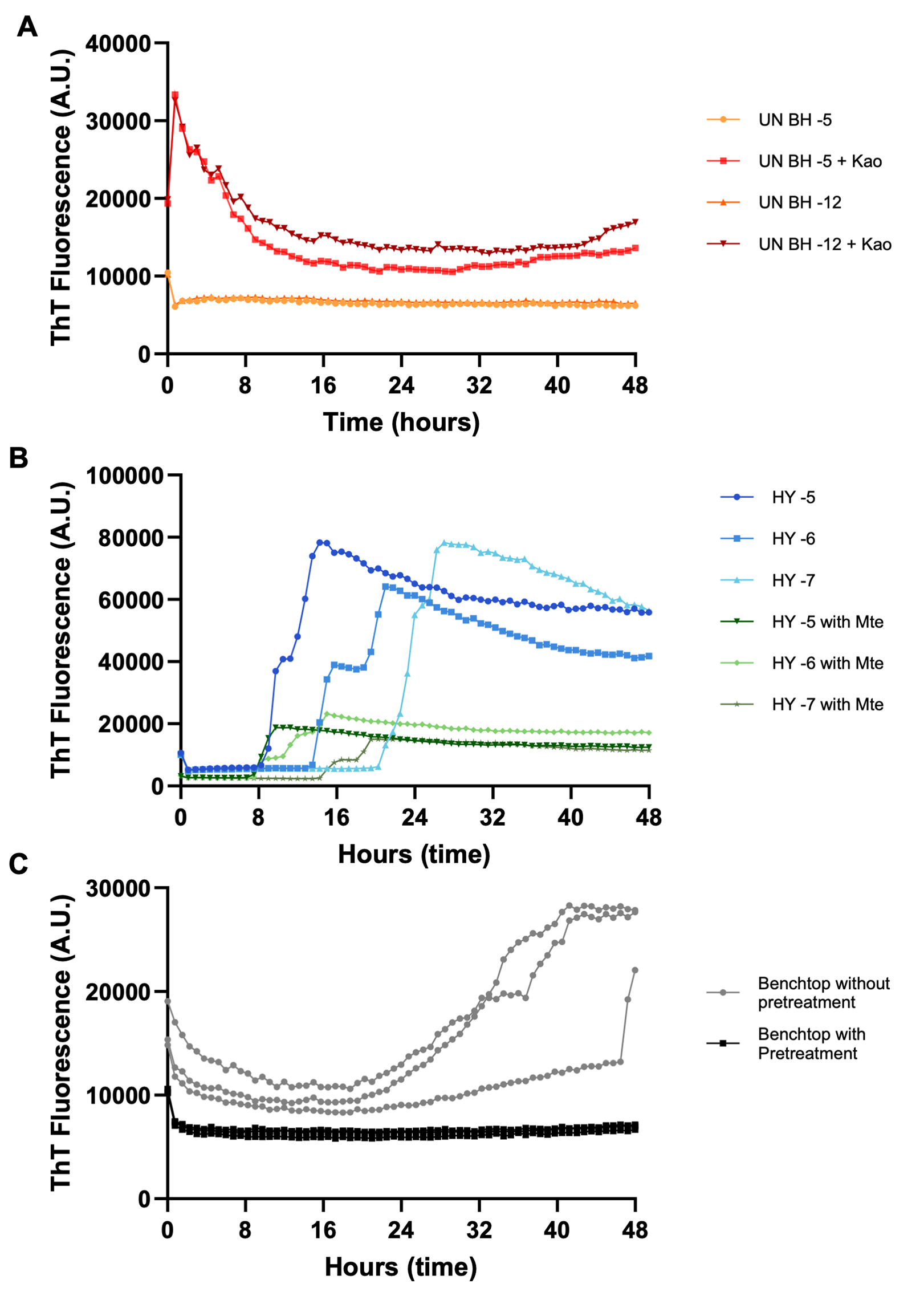

Supplement: Figure S7 — Addition of environmental contamination impacts RT-QuIC. [file msphere.00504-24-s0007.tiff]
