# Supplementary material for: Rapid and sensitive determination of residual prion infectivity from prion-decontaminated surfaces
Source: mSphere. 2024 Aug 27;9(9):e00504-24. doi: 10.1128/msphere.00504-24 (PMC11423590; doi:10.1128/msphere.00504-24)
Supplement: Legends — Supplemental figure legends. [file msphere.00504-24-s0008.docx]

**Figure S1. Dilution preparation.** A) Preparation of positive control dilutions for RT-QuIC evaluation of vertical dilution swab extracts B. Preparation of swab extract samples for RT-QuIC evaluation of vertical dilution swab extracts C. Preparation of swab extract samples for RT-QuIC evaluation of horizontal dilution swab extracts. Created with [BioRender.com](https://biorender.com/).

**Figure S2. Bleach is an effective disinfectant for HY contaminated stainless steel surfaces.** A). RT-QuIC detection for stainless steel surface recovered negative controls included: UN HA BH 10^-3^, UN HA BH 10^-10^ and DPBS. B,C,D). RT-QuIC detection for swab extracts from HY contaminated stainless steel surfaces treated with H_2_O, 70% Ethanol, or undiluted bleach for 10 minutes. Negative plate controls include blank (tissue dilution solution) and uninfected brain homogenate 10^-3^. Each point represents the average MPR from one biological replicate. A positive fluorescence threshold (illustrated by red line) was determined to be at 2. The maxpoint ratio (MPR) reported is the ratio of the maximum fluorescence to the initial fluorescence reading obtained by the plate reader (mean +/- standard deviation).

**Figure S3. Bleach is an effective disinfectant for HY contaminated benchtop surfaces.** A). RT-QuIC detection for benchtop surface recovered negative controls included: UN HA BH 10^-3^, UN HA BH 10^-10^ and DPBS. B,C,D). RT-QuIC detection for swab extracts from HY contaminated benchtop treated with H_2_O, 70% Ethanol, or undiluted bleach for 10 minutes. Negative plate controls include blank (tissue dilution solution) and uninfected brain homogenate 10^-3^. Each point represents the average MPR from one biological replicate. A positive fluorescence threshold (illustrated by red line) was determined to be at 2. The maxpoint ratio (MPR) reported is the ratio of the maximum fluorescence to the initial fluorescence reading obtained by the plate reader (mean +/- standard deviation).

**Figure S4. Bleach is an effective disinfectant for HY contaminated glass surfaces.** A). RT-QuIC detection for glass surface recovered negative controls included: UN HA BH 10^-3^, UN HA BH 10^-10^ and DPBS. B,C,D). RT-QuIC detection for swab extracts from HY contaminated glass surfaces treated with H_2_O, 70% Ethanol, or undiluted bleach for 10 minutes. Negative plate controls include blank (tissue dilution solution) and uninfected brain homogenate 10^-3^. Each point represents the average MPR from one biological replicate. A positive fluorescence threshold (illustrated by red line) was determined to be at 2. The maxpoint ratio (MPR) reported is the ratio of the maximum fluorescence to the initial fluorescence reading obtained by the plate reader (mean +/- standard deviation).

**Figure S5. Impact of montmorillonite on RT-QuIC detection of HY dilutions is dose dependent**. A) RT-QuIC detection of HY dilutions prepared in tissue dilution solution with 10 mg/ml of montmorillonite (Mte). B) RT-QuIC detection of HY dilutions prepared in tissue dilution solution with 1 mg/ml of Mte. C) RT-QuIC detection of HY dilutions prepared in tissue dilution solution with 0.1 mg/ml of Mte. Negative plate controls include blank and uninfected brain homogenate 10^-5^ and 10^-12^ with and without soil. A positive plate control consisted a HY dilution series prepared in standard tissue dilution solution. A positive fluorescence threshold (illustrated by red line) was determined to be at 2. The maxpoint ratio reported is the ratio of the maximum fluorescence to the initial fluorescence reading obtained by the plate reader. Each point represents the average MPR from one biological replicate (mean +/- standard deviation).

**Figure S6. Impact of Hectorite on RT-QuIC detection of HY dilutions is dose dependent.** A) RT-QuIC detection of HY dilutions prepared in tissue dilution solution with 10 mg/ml of hectorite (Hte). B) RT-QuIC detection of HY dilutions prepared in tissue dilution solution with 1 mg/ml of Hte. C) RT-QuIC detection of HY dilutions prepared in tissue dilution solution with 0.1 mg/ml of Hte. Negative plate controls include blank and uninfected brain homogenate 10^-5^ and 10^-12^ with and without soil. A positive plate control consisted a HY dilution series prepared in standard tissue dilution solution. A positive fluorescence threshold (illustrated by red line) was determined to be at 2. The maxpoint ratio reported is the ratio of the maximum fluorescence to the initial fluorescence reading obtained by the plate reader. Each point represents the average MPR from one biological replicate (mean +/- standard deviation).

**Figure S7. Addition of environmental contamination impacts RT-QuIC.** A) Kinetic curves for UN BH 10^-5^ and 10^-12^ dilutions with and without 100mg/ml kaolinite added to the tissue dilution solution. B) Kinetic curves for HY dilutions with and without 1 mg/ml of montmorillonite added to the tissue dilution solution. C) Kinetic curves for swab extracts from uncontaminated benchtop surfaces with or without pretreatment (wiping surface clean with H_2_O).
